# Suppression of telomerase reverse transcriptase (hTERT) expression in differentiated HL-60 cells: regulatory mechanisms

**DOI:** 10.1038/sj.bjc.6690480

**Published:** 1999-06

**Authors:** D Xu, A Gruber, M Björkholm, C Peterson, P Pisa

**Affiliations:** Department of Medicine, Division of Hematology, and Department of Oncology, Radiumhemmet, Karolinska Hospital, Stockholm, SE-171 76, Sweden; Department of Medicine and Care, Clinical Pharmacology, Faculty of Health Science, Linköping University, Linköping, SE-581 85, Sweden

**Keywords:** hTERT, telomerase, HL-60 cells, differentiation

## Abstract

Telomerase activity, associated with cellular immortalization and tumorigenesis, is suppressed during terminal differentiation of HL-60 promyelocytic leukaemic cells. However, it is poorly understood how telomerase activity is regulated in differentiated HL-60 cells. In the present study, we demonstrate that the down-regulation of telomerase reverse transcriptase (hTERT) expression, the catalytic subunit, occurs prior to the suppression of telomerase activity in differentiated HL-60 cells. In contrast, the expression of telomerase RNA template (hTR) and telomerase associated protein (TP1) is not reduced. This down-regulation of hTERT expression is achieved through inhibition of gene transcription, in which process new protein synthesis is required. Moreover, the rapid down-regulation of hTERT expression followed by the inhibition of telomerase activity is a specific component of the differentiation programme and not simply a consequence of cell cycle arrest. Serum-deprivation of HL-60 cells causes cell cycle arrest without differentiation and this does not result in a significant reduction in hTERT mRNA levels within the first 24 h. Our findings suggest that hTERT expression is stringently controlled at transcriptional level in HL-60 cells. The downregulation of hTERT expression in the HL-60 cell differentiation model may represent a general regulatory mechanism through which telomerase becomes repressed during development and differentiation of human somatic cells. © 1999 Cancer Research Campaign


*British Journal of Cancer* (1999) **80**(8), 1156–1161
© 1999 Cancer Research Campaign
Article no. bjoc.1999.0480

### Suppression of telomerase reverse transcriptase (hTERT) expression in differentiated HL-60 cells: regulatory mechanisms

**D Xu[1], A Gruber[1], M Björkholm[1], C Peterson[3] and P Pisa[2]**

[1]Department of Medicine, Division of Hematology and [2]Department of Oncology, Radiumhemmet, Karolinska Hospital, SE-171 76 Stockholm, Sweden; [3]Department of Medicine and Care, Clinical Pharmacology, Faculty of Health Sciences, Linköping University, SE-581 85 Linköping, Sweden

**Summary** Telomerase activity, associated with cellular immortalization and tumorigenesis, is suppressed during terminal differentiation of HL-60 promyelocytic leukaemic cells. However, it is poorly understood how telomerase activity is regulated in differentiated HL-60 cells. In the present study, we demonstrate that the down-regulation of telomerase reverse transcriptase (hTERT) expression, the catalytic subunit, occurs prior to the suppression of telomerase activity in differentiated HL-60 cells. In contrast, the expression of telomerase RNA template (hTR) and telomerase associated protein (TP1) is not reduced. This down-regulation of hTERT expression is achieved through inhibition of gene transcription, in which process new protein synthesis is required. Moreover, the rapid down-regulation of hTERT expression followed by the inhibition of telomerase activity is a specific component of the differentiation programme and not simply a consequence of cell cycle arrest. Serum-deprivation of HL-60 cells causes cell cycle arrest without differentiation and this does not result in a significant reduction in hTERT mRNA levels within the first 24 h. Our findings suggest that hTERT expression is stringently controlled at transcriptional level in HL-60 cells. The downregulation of hTERT expression in the HL-60 cell differentiation model may represent a general regulatory mechanism through which telomerase becomes repressed during development and differentiation of human somatic cells.

**Keywords**: hTERT; telomerase; HL-60 cells; differentiation

Human telomerase, a multicomponent ribonucleoprotein enzyme that extends chromosome ends with (TTAGGG)n telomeric repeat sequences, is generally inactive in human normal somatic cells but active in a variety of human tumour cell lines and primary malignant tissues (Harley et al, 1994; Kim et al, 1994). Activation of telomerase has been implicated in cell immortalization and tumorigenesis. Telomerase is thus a potentially attractive target for cancer therapy.

We and others (Albanell et al, 1996; Bestilny et al, 1996; Holt et al, 1996; Xu et al, 1996) have demonstrated that the suppression of telomerase activity in promyelocytic leukaemic cells follows terminal differentiation induced by various differentiating agents. However, the mechanisms of telomerase inactivation during differentiation of HL-60 cells remain unknown. Only lately is our understanding of the regulation of telomerase activity starting to emerge thanks to the identification of the human telomerase subunits (Feng et al, 1995; Harrington et al, 1997; Kilian et al, 1997; Meyerson et al, 1997; Nakamura et al, 1997). Telomerase subunits RNA template (hTR) and telomerase associated protein (TP1) were found largely unchanged or even increased in differentiated promyelocytic leukaemic cell lines (Meyerson et al, 1997; Reichman et al, 1997). These results are in accordance with the observation that hTR and TP1 are expressed both in normal and in malignant human cells and that their expression levels do not correlate with the biological activity of telomerase (Feng et al, 1995; Meyerson et al, 1997; Nakamura et al, 1997). Most recently, the RNA encoding the human telomerase catalytic component called telomerase reverse transcriptase (hTERT) has been cloned (Kilian et al, 1997; Meyerson et al, 1997; Nakamura et al, 1997). Accumulated data support the notion that the expression of hTERT is a rate-limiting step in the control of telomerase activity. Telomerase activity can be reconstituted by the ectopic expression of hTERT in human normal telomerase-negative fibroblasts (Bodnar et al, 1998). A down-regulation of hTERT expression has been observed in HL-60 cells induced to differentiate by treatment with all-*trans* retinoic acid (ATRA) (Meyerson et al, 1997).

The present work was undertaken to further evaluate alterations in telomerase activity, as well as hTERT, hTR and TP1 expression during terminal differentiation of HL-60 cells, and especially address the regulatory mechanisms of hTERT expression in this model.

### MATERIALS AND METHODS

### Chemicals and cell culture

ATRA, actinomycin D (ACD) and cycloheximide (CHX) were purchased from Sigma (St Louis, MO, USA), dimethyl sulphoxide (DMSO) from Merck (Darmstadt, Germany) and dihydroxyvitamin $D_3$ ($VD_3$) from Abbott Laboratories (North Chicago, IL,

*Received 28 August 1998*
*Revised 6 January 1999*
*Accepted 7 January 1999*

*Correspondence to:* D Xu

USA). HL-60 cells were grown in RPMI-1640 medium (Life Technologies, Paisley, UK) supplemented with 10% fetal calf serum (FCS), 100 U $ml^{-1}$ penicillin and 2 mM L-glutamine. For the induction of differentiation, the cells at 0.25–0.5 × $10^6$ per ml were treated with 2 × $10^{-6}$ M ATRA, or 5 × $10^{-7}$ M $VD_3$, or 1.25% v/v DMSO and harvested at various time points. Viable cells always exceeded 90% as determined by the trypan blue exclusion dye test. For serum starvation, HL-60 cells were washed twice with phosphate-buffered saline (PBS) and then incubated in the same medium but without FCS.

### Assays for differentiation

CD11b antigen expression was chosen as the specific marker for HL-60 cell differentiation towards both granulocytes and monocytes. CD11b antigen determination and the isolation of CD11b-positive and -negative cells have been described in detail elsewhere (Xu et al, 1996).

### Telomerase activity assay

A commercial telomerase polymerase chain reaction (PCR) enzyme-linked immunosorbent assay (ELISA) kit (Boehringer Mannheim, Stockholm), based on telomeric repeat amplification protocol (TRAP) introduced by Kim et al (1994), was used to determine telomerase activity in control and differentiating agents-treated HL-60 cells according to the manufacturer's protocol during 25 PCR cycles (Xu et al, 1998). Protein extraction and measurement was performed as described previously (Xu et al, 1996).

### RNA extraction and reverse transcription

Total cellular RNA was extracted by using the ULTRASPEC™– II RNA kit (Biotecx Lab., Houston, TX, USA). To purify nuclear RNA, cells were first suspended in ice-cold NP-40 containing lysis buffer (10 mmol Tris, pH 7.4, 10 mmol sodium chloride, 3 mmol magnesium chloride and 0.5% NP-40), centrifuged at 800 ***g*** for 5 min and washed three times with the same buffer. The isolated nuclei were then subjected to RNA extraction with the above kit. RNA yield and purity were determined spectrophotometrically at 260–280 nm (Perkin-Elmer, Lambda Bio) and the integrity of RNA verified by electrophoretic size separation in 1% ethidium bromide-stained agarose gels. cDNA was synthesized using random primers (N6) (Pharmacia, Uppsala, Sweden) and MMLV reverse transcriptase, as described earlier (Xu et al, 1998).

### Quantitative determination of hTERT, hTR and TP1 expression by competitive RT-PCR

PCR primer sequences and conditions were described by Nakamura et al (1997). The competitive templates of hTERT, hTR and TP1 fragments, constructed by mimic PCR, were 21 bp longer than their wild-type counterparts. cDNA corresponding to 50 ng of RNA was co-amplified with appropriate competitors (5 × $10^3$, $10^4$ and $10^3$ molecules for hTERT, hTR and TP1 respectively) in 25 μl of reaction mixture. PCR was performed using 32, 31 and 31 cycles for hTERT, hTR and TP1 respectively. In addition, $\beta_2$-microglobulin ($\beta_2$M) expression was used as a control for RNA loading and RT efficiency and co-amplified with its competitors (2.5 × $10^4$ molecules) with the following RNA-specific primers

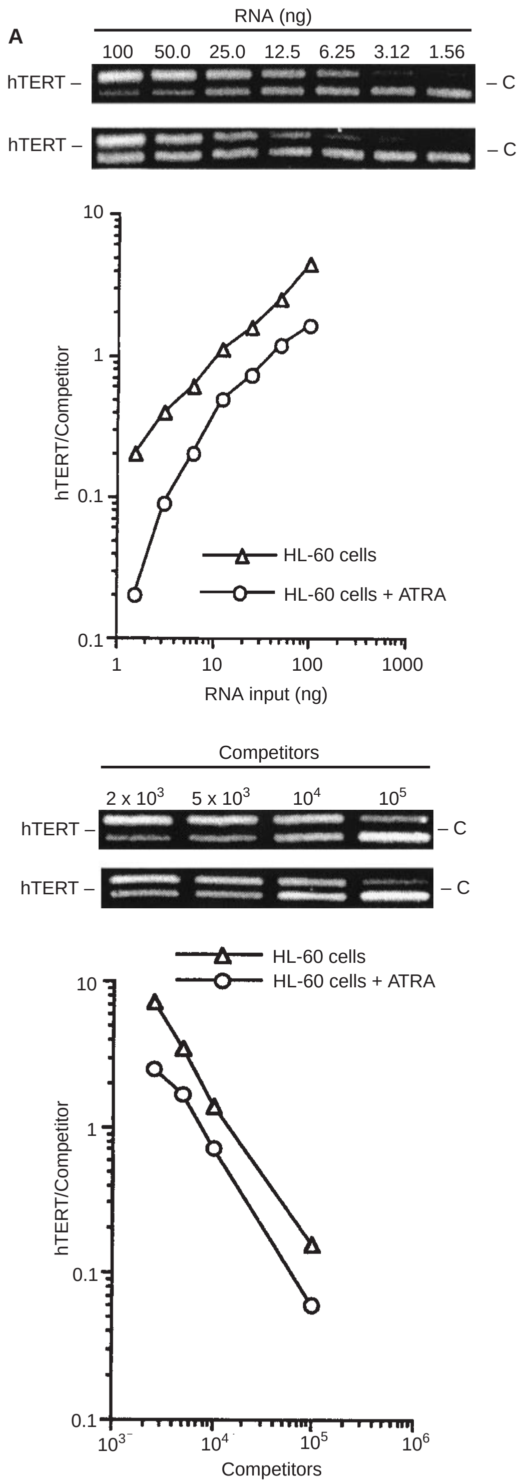

**Figure 1** Validation of quantitative detection of hTERT mRNA by using competitive RT-PCR. (**A**) Serial dilutions of RNA derived from untreated (upper) and ATRA-treated HL-60 cells (lower) for 8 h were coamplified with the fixed number of competitors (5 × $10^3$ molecules). The resulting PCR products were resolved in 4% of Metaphor gels stained with ethidium bromide and photographed. The images were analysed by densitometry. The signal intensity ratio of hTERT mRNA to the competitor was plotted against the amount of RNA present in each lane. (**B**) The fixed amounts of total RNA (50 ng) derived from untreated (upper) and ATRA-treated HL60 cells (lower) for 8 h were co-amplified with the different numbers of competitors and the ratio of hTERT mRNA to the competitor was plotted against the number of competitors present in each lane. C: Competitor

*© 1999 Cancer Research Campaign*

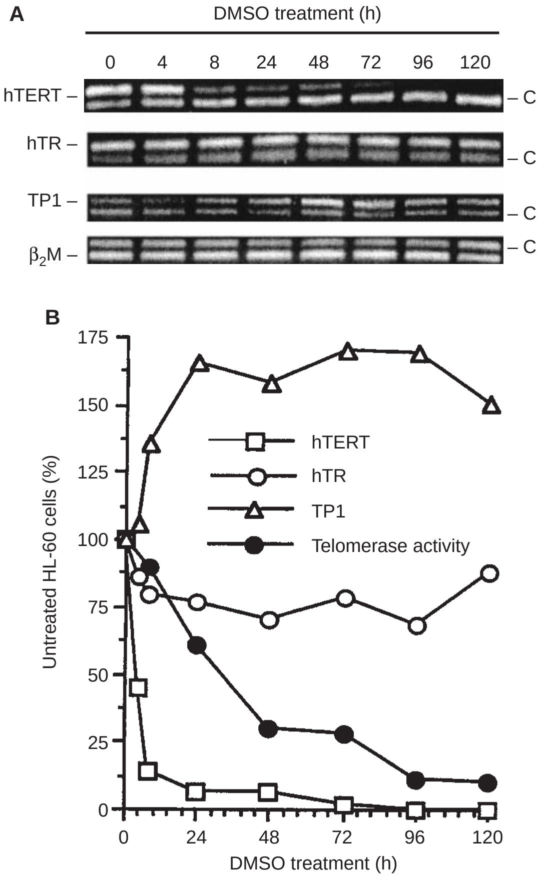

**Figure 2** Telomerase activity and its subunits hTERT, hTR and TP1 expression in HL-60 cells treated with DMSO. (**A**) hTERT, hTR, TP1 and $\beta_2$M cDNA were co-amplified with their competitors, respectively, and PCR products were resolved in 4% Metaphor gels stained with ethidium bromide. C: Competitor. (**B**) The ethidium bromide-stained PCR products shown in panel **A** were photographed and the images analysed by densitometry. The intensity ratio of the target mRNA to the competitor normalized to the ratio of $\beta_2$M/Competitor in each lane was expressed as the percentage of that in untreated HL-60 cells. Telomerase activity at each time point, determined by using telomerase PCR–ELISA kit, was expressed as the percentage of that in untreated HL-60 cells. Data shown is one representative of two separate experiments

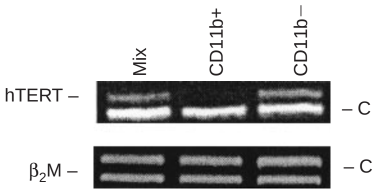

**Figure 3** hTERT expression in CD11b+ and CD11b− HL-60 cells treated with $VD_3$. The cells were incubated with $5 \times 10^{-7}$ µM of $VD_3$ for 72 h and CD11b+ and CD11b+ cells were separated by using Mini-MAX. Total cellular RNA was extracted and subjected to competitive RT-PCR analysis for hTERT expression. C: Competitor

(5′-primer: GAATTGCTATGTGTCTGGGT; 3′-primer: CATCTT-CAAACCTCCATGATG) during 25 cycles (94°C for 30 s, 60°C for 30 s and 72°C for 60 s) (Xu et al, 1998). PCR products were resolved in 4% Metaphor agarose gels stained with ethidium bromide, visualized in UV light and photographed. Volumetric integration of signal intensities was performed by using the NIH image software (Version 1.58). The relative levels of hTERT, hTR and TP1 mRNA were given by the ratio of the target to competitor signal intensity and normalized to the ratio of $\beta_2$M to its competitor.

### Determination of cell cycle distribution by flow cytometry

By using the Cycle TEST™ PLUS DNA Reagent kit (Becton-Dickinson, San José, CA, USA) DNA profile was determined in untreated and treated HL-60 cells. Briefly, $1 \times 10^6$ cells were treated with trypsin and RNAase, stained in propidium iodide and evaluated by flow cytometry (Becton-Dickinson). The cell percentage in G0/G1, S and G2/M phases was calculated by using Mod Fit LT for Mac V2 software (Becton-Dickinson).

### RESULTS AND DISCUSSION

### Validation of quantitative RT-PCR for hTERT, hTR and TP1 expression

As shown in Figure 1A, the signal ratio of the hTERT and co-amplified competitor PCR products, with a less than 20% coefficient variation (CV), corresponded well to the dilution factor of RNA derived from HL-60 cells. A linearity of hTERT assay was also demonstrated at the fixed amount of RNA input with different numbers of competitors (Figure 1B). Similar results were obtained with hTR and TP1 quantification (data not shown).

### Differential regulation of telomerase subunit expression during differentiation of HL-60 cells

As reported previously, differentiating-agents DMSO, ATRA and $VD_3$ induce HL-60 cells to undergo terminal differentiation which was accompanied by the suppression of telomerase activity (Holt et al, 1996; Xu et al, 1996; Meryerson et al, 1997).

Since telomerase is a multicomponent reverse transcription enzyme, the control of telomerase activity may operate at multiple levels. So far, no studies have addressed the gene expression levels of all three telomerase subunits during terminal differentiation of HL-60 cells. Moreover, previous reports produced conflicting results with regard to hTR expression in differentiated promyelocytic leukaemic cells (Bestilny et al, 1996; Meyerson et al, 1997). The extended evaluation of the gene expression of all three telomerase components, hTERT, hTR and TP1 was performed on HL-60 cells treated with differentiating agents. In the presence of DMSO or ATRA, the level of hTERT mRNA declined rapidly; only less than 10% of the control level remained after 24 h (Figure 2). Clearly, the down-regulation of hTERT expression is an early event in differentiated HL-60 cells. Compared to DMSO or ATRA-treated cells, a relatively high level of hTERT still remained in $VD_3$-treated HL-60 cells after 120 h (data not shown). The explanation for this discrepancy is that $VD_3$ treatment induces differentiation in 60–70% of HL-60 cells and that remaining undifferentiated HL-60 cells constitutively express hTERT mRNA. This was documented in Figure 3, showing that hTERT expression remained at a high level in undifferentiated CD11b− cells but was hardly detectable in CD11b+ cells after the exposure of HL-60

*© 1999 Cancer Research Campaign*

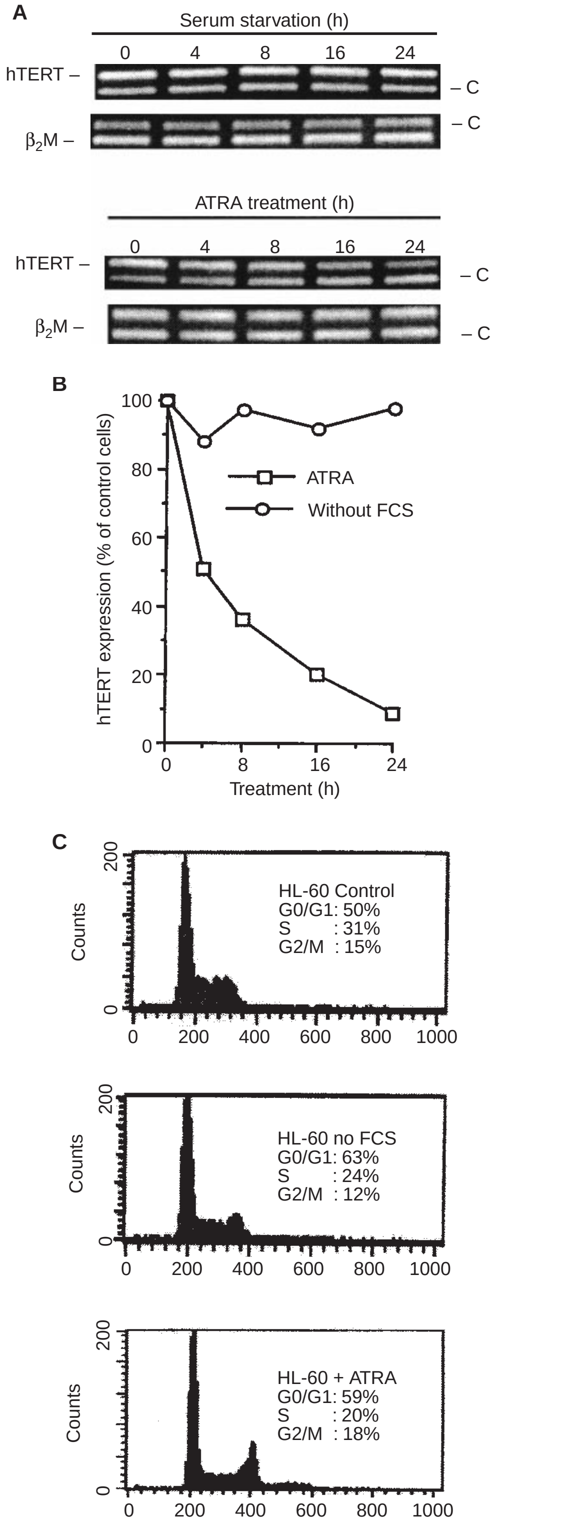

**Figure 4** hTERT expression and cell cycle distribution in ATRA-treated and serum-deprived HL-60 cells. Exponentially growing cells were treated with 2 μM ATRA or serum-starved for 24 h and harvested for RNA extraction and cell cycle analysis. (**A**) Expression of hTERT mRNA in ATRA-treated and serum-deprived HL60 cells. C: Competitor. (**B**) The ethidium bromide-stained PCR products shown in panel **A** were photographed and the images were analysed by densitometry. The signal intensity ratio of hTERT mRNA to the competitor normalized to the ratio of $\beta_2$M/Competitor in each lane was expressed as the percentage of that in untreated HL-60 cells. (**C**) DNA content flow cytometric profiles in ATRA-treated and serum-deprived HL-60 cells for 24 h. The experiments were performed twice with similar results

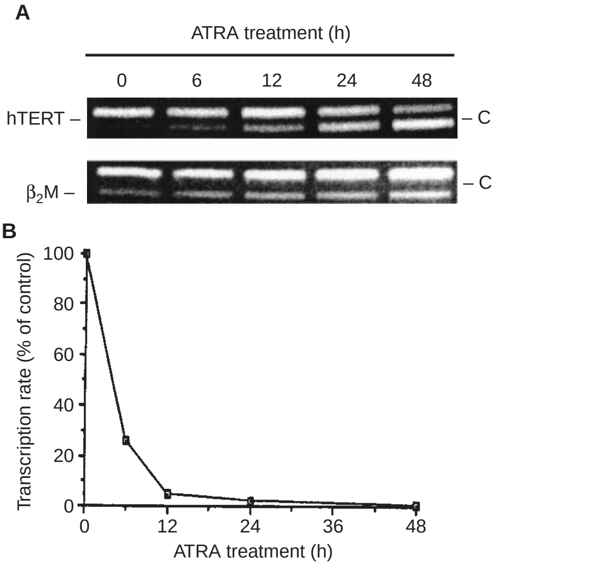

**Figure 5** The transcription rate of hTERT gene in ATRA-treated HL60 cells determined by using competitive RT-PCR. (**A**) Nuclear RNA was extracted and subjected to RT-PCR with hTERT competitors. $\beta_2$M was coamplified with its competitors. C: Competitor. (**B**) The transcription rate of hTERT gene was expressed as the percentage of that in untreated HL-60 cells according to the signal intensity ratio of hTERT/competitor. The analysis was performed twice with similar results cells to $VD_3$ for 72 h. The data indicate that the down-regulation of hTERT expression during the terminal differentiation of HL-60 cells is a differentiation-related event. Consistent with this, when another myeloid leukaemic cell line, K562 cells, known not to be terminally differentiated by treatment with ATRA, were incubated with 2 μM of ATRA, there was no detectable change in hTERT mRNA levels within 96 h (data not shown).

In contrast, up to 70% increase in TP1 mRNA was observed in HL-60 cells incubated with all three differentiating agents (Figure 2). hTR expression levels of differentiated HL-60 cells remained largely unchanged as compared to the control cells (Figure 2).

The observation that suppression of telomerase activity is accompanied by down-regulation of hTERT mRNA, but not hTR and TP1 expression in differentiated HL-60 cells supports the notion that hTERT is the rate-limiting determinant of telomerase activity. Down-regulation of hTERT expression in HL-60 cell differentiation model may represent a general regulatory pathway through which telomerase becomes inactivated during development and differentiation of human somatic cells. Unlike hTERT, the expression of hTR is loosely controlled and is up-regulated in many human malignant and normal cell types with a correlation to active proliferation status, but not telomerase activity (Greider, 1998). A recent investigation (Beattie et al, 1998) showed that the coexpression of hTERT protein and hTR is capable of reconstituting enzyme activity of telomerase in vitro, suggesting that the core enzyme complex may consist of only two components, hTERT and hTR. Taken together, TP1 expression may be redundant in terms of the regulation of telomerase activity.

### Regulatory mechanisms of hTERT expression in differentiated HL-60 cells

Since hTERT expression is the determinant for the control of telomerase activity and it has been shown that the induction of

*© 1999 Cancer Research Campaign*

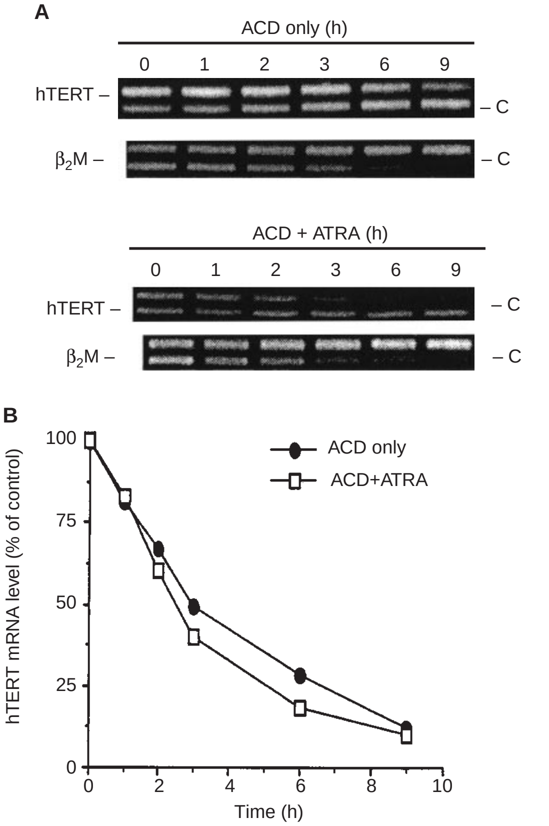

**Figure 6** Comparison of the half-life of hTERT transcripts in control and ATRA-treated HL-60 cells. New RNA synthesis was blocked by ACD and the half-life of hTERT mRNA was estimated with competitive RT-PCR. One representative of three separate experiments was shown with a half-life of hTERT mRNA 3 h (median) for control HL-60 cells and 2.6 h (median) for ATRA-treated HL-60 cells (**A** and **B**). C: Competitor

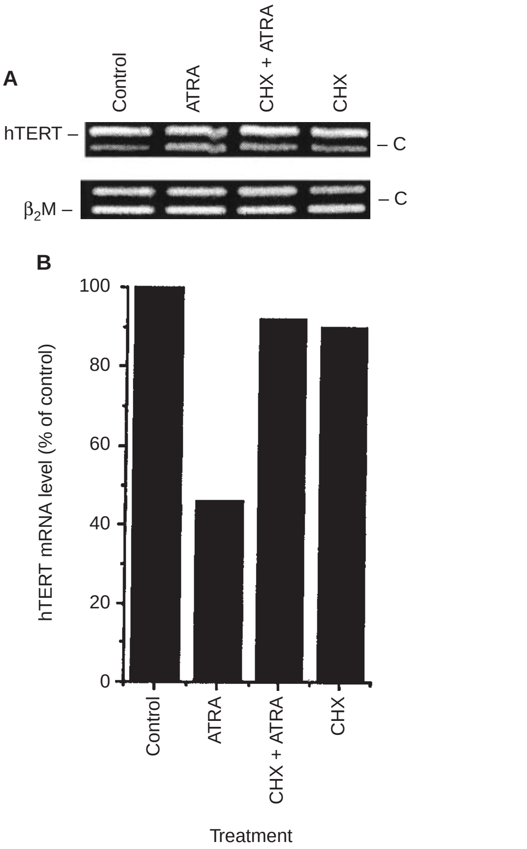

**Figure 7** Effects of CHX on the down-regulation of hTERT mRNA in ATRA-treated HL-60 cells. The cells were incubated with CHX or CHX plus ATRA for 4 h to inhibit new protein synthesis. Then hTERT expression was quantitatively analysed by competitive RT-PCR. One of three separate experiments with similar results was shown (**A** and **B**). C: Competitor hTERT expression is required for telomerase activation during cellular immortalization and tumour progression, elucidation of the regulatory mechanisms of hTERT expression will certainly contribute to a better understanding of telomerase activity control at the molecular level.

Some recent observations have suggested that telomerase activity is growth-regulated, high in the actively cycling culture and low in the quiescent cells (Greider, 1998). As differentiated HL-60 cells eventually exit out of cell cycle, we thus ask whether the down-regulation of telomerase activity and hTERT expression is simply related to the cellular proliferation status. As shown in Figure 4, ATRA-treated HL-60 cells exhibited a rapid decline in hTERT expression and a slight cell cycle arrest within 24 h. On the contrary, 24 h of serum starvation of HL-60 cells had a similar effect on the cell cycle arrest as ATRA-treatment, but there was no clear change in hTERT mRNA level during this period (Figure 4). A significant down-regulation of hTERT expression in serum-deprived HL-60 cells was observed after 48 h of incubation without FCS (data not shown). It is evident from the present data that the differentiation-triggered down-regulation of hTERT expression in HL-60 cells is a more active process, not simply a consequence of cell cycle arrest.

To determine whether hTERT transcripts are modulated in differentiated HL-60 cells due to alteration in transcriptional activity in nuclei, nuclear RNA was isolated and the levels of nascent hTERT mRNA was measured by competitive RT-PCR, a method which gives identical results as nuclei run-off test, a classical assay for transcription rate of gene expression (Herzog et al, 1993). As seen in Figure 5, the transcription rate of hTERT declined rapidly in ATRA-treated HL-60 cells and only a very low level of activity was recorded after 24 h. $\beta_2$M as an internal control for loading amounts of nuclear RNA was analysed in parallel on nuclear RNA.

We then asked whether post-transcriptional regulation affects hTERT mRNA levels. To compare the stability of hTERT transcripts between untreated and ATRA-treated HL-60 cells, analysis of the hTERT mRNA half-life was performed by incubating HL-60 cells with ACD at 10 μg ml$^{-1}$ and purifying RNA at the indicated time points. New RNA synthesis was blocked by ACD, which was demonstrated by almost no $^3$H-thymidine incorporated into HL-60 cells in the presence of 10 μg ml$^{-1}$ of ACD. HL-60 cells had been exposed to ATRA for 3 h prior to the addition of ACD. This caused a lower basic expression level of hTERT mRNA at time 0 compared to the control sample. The half-life of hTERT mRNA in the control HL-60 cells ranged between 2.6 and 3.8 h (median 3 h) in three experiments, whereas that of ATRA-treated HL-60 cells was 2.4–3.2 (median 2.6 h) (Figure 6). The difference in the stability of hTERT mRNA was not significant, but it is likely that the slightly shortened half-life of hTERT transcripts in differentiated HL-60 cells partially results in decline

*© 1999 Cancer Research Campaign*

in the levels of hTERT mRNA. The short half-life of hTERT transcripts provides the explanation for the rapid reduction of hTERT mRNA and delay in the decline of telomerase activity with a longer half-life seen in differentiated HL-60 cells.

Finally, we wanted to address the question whether ongoing protein synthesis was required for differentiation-triggered down-regulation of hTERT expression. New protein synthesis was fully blocked in the presence of 100 μg $ml^{-1}$ of CHX, an inhibitor of protein synthesis (Matikainen et al, 1996). HL-60 cells were incubated with CHX at 100 μg $ml^{-1}$ for 20 min first, and thereafter ATRA or DMSO was added to the culture for up to 4 h. Quantitative RT-PCR analysis showed that the downregulation of hTERT transcripts induced by ATRA was blocked in the presence of CHX (Figure 7). A similar result was obtained in DMSO plus CHX-treated HL-60 cells (data not shown). These observations suggest that new protein(s) are involved in the hTERT mRNA modulation process.

In summary, the suppression of telomerase activity is preceded by a down-regulation of hTERT mRNA but unrelated to the expression of hTR and TP1 during differentiation of HL-60 cells. The onset of reduction of hTERT expression during cellular differentiation is at least in part independent of cell proliferation status. We further demonstrate that the down-regulation of hTERT mRNA in differentiated HL-60 cells is achieved through inhibition of its transcription. In these regulatory processes, active protein synthesis is required and the newly synthesized protein(s) participate in either transcription inhibition or controlling of the steady-state level of hTERT mRNA. These findings provide new insights into regulatory control of telomerase activity at the molecular level and may be useful for future development of anti-telomerase strategy against human malignancies.

### ACKNOWLEDGEMENTS

This work was supported by the Swedish Cancer Society, the Cancer Society in Stockholm, Karolinska Institute Funds and the King Gustaf Vth Jubilee Fund. PP is a research fellow of the Swedish Medical Research Council.

### REFERENCES

Albanell J, Han W, Mellado B, Gunawardane R, Scher H and Dmitrovsky E (1996) Telomerase activity is repressed during differentiation of maturation-sensitive but not resistant human tumor cell lines. *Cancer Res* **56**: 1503–1508

Beattie TL, Zhou W, Robinson MO and Harrington L (1998) Reconstitution of telomerase activity in vitro. *Curr Biol* **8**: 177–180

Bestilny L, Brown C, Miura Y, Robertson L and Riabowol K (1996) Selective inhibition of telomerase activity during terminal differentiation of immortal cell lines. *Cancer Res* **56**: 3796–3802

Bodnar A, Ouellette M, Frolkis M, Holt S, Chiu C, Morin G, Harley C, Shay J, Lichtsteiner S and Wright W (1998) Extension of life-span by introduction of telomerase into normal human cells. *Science* **279**: 349–352

Feng L, Funk W, Wang S-S, Weinrich S, Avillion A, Chiu C, Adams R, Chang E, Allsopp R, Yu J, Le S, West M, Harley C, Andrews W, Greider C and Villeponteau B (1995) The RNA component of human telomerase. *Science* **269**: 1236–1241

Greider CW (1998) Telomerase activity, cell proliferation, and cancer. *Proc Natl Acad Sci USA* **95**: 90–92

Harley CB, Kim NW, Prowse KR, Weinreich SL, Hirsch KS, West MD, Bacchetti S, Hirte HW, Counter CM, Greider CW, Piatyszek MA, Weight WE and Shay JW (1994) Telomerase, cell immortality, and cancer. *Cold Spring Harbor Symp Quant Biol* **59**: 307–315

Harrington L, Mcphail T, Mar V, Zhou W, Oulton R, Bass M, Arruda I and Robinson M (1997) A mammalian telomerase-associated protein. *Science* **275**: 973–977

Herzog C, Tsokos M, Bates S and Fojo A (1993) Increased mdr-1/p-glycoprotein expression after treatment of human colon carcinoma cells with p-glycoprotein antagonists. *J Biol Chem* **268**: 2946–2952

Holt SE, Wright WE and Shay JW (1996) Regulation of telomerase activity in immortal cell lines. *Mol Cell Biol* **16**: 2932–2939

Kilian A, Bowtell DDL, Abud HE, Hime GR, Venter DJ, Keese PK, Duncan EL, Reddel RR and Jefferson RA (1997) Isolation of a candidate human telomerase catalytic subunit gene, which reveals complex splicing patterns in different cell types. *Hum Mol Genet* **12**: 2011–2019

Kim N, Ma MP, Prowse K, Harley C, West M, Ho P, Coviello G, Wright W, Weinrich S and Shay J (1994) Specific association of human telomerase activity with immortal cells and cancer. *Science* **266**: 2011–2015

Matikainen S, Ronni T, Hurme M, Pine R and Julkunen (1996) Retinoic acid activates interferon regulatory factor-1 gene expression in myeloid cells. *Blood* **88**: 114–123

Meyerson M, Counter C, Eaton E, Ellisen L, Steiner P, Caddle S, Ziaugra L, Beijersbergen R, Davidoff M, Liu Q, Bacchetti S, Haber D and Weinberg R (1997) hEST2, the putative human telomerase catalytic subunit gene, is up-regulated in tumor cells and during immortalization. *Cell* **90**: 785–795

Nakamura T, Morin G, Chapman K, Weinrich S, Andrews W, Lingner J, Harley C and Cech T (1997) Telomerase catalytic subunit homologs from fission yeast and human. *Science* **277**: 955–959

Reichman T, Albanell J, Wang X, Moore M and Studzinski G (1997) Downregulation of telomerase activity in HL60 cells by differentiating agents is accompanied by increased expression of telomerase-associated protein. *J Cell Biochem* **67**: 13–23

Xu D, Gruber A, Peterson C and Pisa P (1996) Suppression of telomerase activity in HL60 cells after treatment with differentiating agents. *Leukemia* **10**: 1354–1357

Xu D, Gruber A, Peterson C and Pisa P (1998) Telomerase activity and the expression of telomerase components in acute myelogenous leukaemia. *Br J Haematol* **102**: 1365–1373.
